# Mapping Relation between Contour Error Components of Crankshaft Pin Journal and Axis Position Control Error of Oscillating Grinding Machine

**DOI:** 10.3390/s21196497

**Published:** 2021-09-29

**Authors:** Xiaoyan Fang, Xiaowei Sheng, Yize Sun, Yang Xu

**Affiliations:** 1 College of Mechanical Engineering, Donghua University, Shanghai 201620, China; fangxy@smtw.com (X.F.); sunyz@dhu.edu.cn (Y.S.); xuyang@dhu.edu.cn (Y.X.); 2 Shanghai Machine Tool Works Ltd., Shanghai 200093, China

**Keywords:** contour error analysis, axis position control error, EEMD, oscillating grinding, crankshaft pin journal

## Abstract

Automatic crankshaft production lines require high reliability and accuracy stability for the oscillating grinding machine. Crankshaft contour error represent the most intuitive data in production field selective inspection. If the mapping relation between the contour error components of the crankshaft pin journal and the axis position control error of the oscillating grinding machine can be found, it would be great significance for the reliability maintenance of the oscillating grinding machine. Firstly, a contour error decomposition method based on ensemble empirical mode decomposition (EEMD) is proposed. Secondly, according to the contour generating principle of the pin journal by oscillating grinding, a calculation method to obtain the effect of the axis position control error of the oscillating grinder on the contour error of the pin journal is proposed. Finally, through the grinding experiments, the error data are acquired and measured to calculate and decompose the contour error by using the proposed methods for obtaining the mapping relation between the crankshaft pin journal contour error and the axis position control error. The conclusions show that the proposed calculation and decomposition methods can obtain the mapping relation between the contour error components of the crankshaft pin journal and the axis position control error of the oscillating grinding machine, which can be used to predict the key functional component performance of the machine tool from the oscillating grinding workpiece contour error.

## 1. Introduction

The crankshaft is a critical component of the automobile engine, and its machining quality directly affects engine performance and reliability. Therefore, crankshaft manufacturing plays a very significant role in the automotive industry. In the mass production of engine crankshafts, the pin chasing grinding technology based on the oscillating grinding machines has been widely implemented to meet machining accuracy and efficiency requirements [1,2]. During the oscillating grinding, the workpiece rotation-axis (C axis) and the transverse feed of grinding carriage-axis (X axis) are executed to keep the grinding wheel tangent with the crankshaft pin journals [3,4,5,6]. The oscillating grinding method is capable of providing higher machining efficiency, more flexibility, and better precision than the traditional grinding method that needs an appropriative fixture to adjust the rotation center repeatedly [1]. Recently, the application of the oscillating grinding machines in the automotive and shipping industry has progressed, and thus the reliability and stability of the oscillating grinding machines continues to grow. According to the characteristics of the oscillating grinding process, the contour error components of the crankshaft pin journal apparently contain error information of the C axis and X axis. The contour errors of the crankshaft pin journal are the most intuitive data in production field selective inspection. If the mapping relation between the contour error components of the crankshaft pin journal and the axis position control error of the oscillating grinding machine can be built, it would be great significance for the reliability maintenance of the oscillating grinding machine.

Engineering surface texture is considered as the “fingerprint” of the manufacturing process [7], where the performance information of the machine tool, the condition information of the cutting tool, and the characteristic information of materials are involved [8]. The oscillating grinding contour error of the crankshaft pin journal, which contains the performance information of the grinder, the condition information of the grinding wheel, and the performance of the crankshaft material, is the fingerprint of the pin chasing grinding process. According to the special characteristics of the pin chasing grinding process, the C axis and X axis have an obvious effect on the contour error of the crankshaft pin journal. Conversely, the crankshaft contour error based on this kind of grinding process apparently covers the performance information of the oscillating grinding machines, especially the performance information of C axis and X axis. There are some traditional contour error analysis methods, such as the Fourier transform and the wavelet transform [8,9,10,11], which are based on the integral transform and required to design or choose the proper basis function according to the signal characteristics of the contour error. These analysis methods strongly depend on the prior knowledge and lack of flexibility and adaptivity in analyzing and processing real and complicated signals. Empirical mode decomposition (EMD), proposed by Huang et al. in 1998 [12], can be used to decompose a complicated signal into a series of intrinsic mode functions (IMFs) and they verified that, even in the worst conditions, the instantaneous frequency of IMFs obtained by Hilbert transform can map into the required physical factors. To solve the mode mixing problem in EMD, an ensemble empirical mode decomposition (EEMD) was presented by Wu and Huang [13]. The EEMD method does not need to design a basis function based on prior knowledge. The decomposition is posterior and completely driven by the data. The EEMD method is an adaptive mode decomposition method and has been widely used in machinery fault diagnosis [14,15]. However, there are no references to contour error analysis using EEMD.

**Figure 1:**
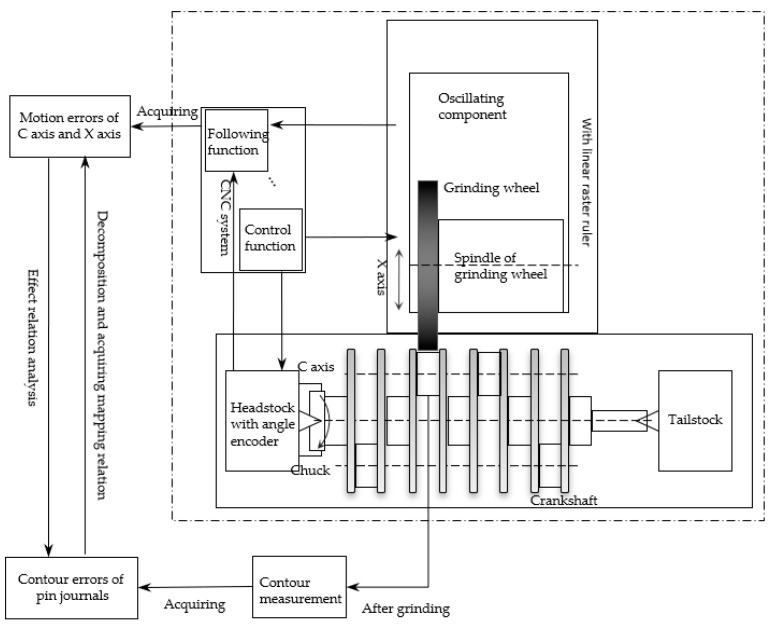
Schematic illustration of an oscillating grinder and the sources of errors.

The schematic of an oscillating grinder and the error sources are illustrated in Figure 1. The contour error of the crankshaft pin journal is measured after oscillating grinding. Computerized numerical control (CNC) system of the crankshaft oscillating grinder can acquire the real-time position information of the axes, which contains position control errors of the C axis and X axis. In this paper, a contour error decomposition method of the crankshaft pin journal based on EEMD is firstly proposed. As the contour error is closed data, periodic extension is applied in both ends for avoiding the boundary effect. According to the contour generating principle of the crankshaft pin journal by oscillating grinding, a calculation method to identify the effect of the axis position control error on the contour error of the crankshaft pin journal is then proposed. Finally, through the grinding experiments, the error data are acquired and measured to calculate and decompose the contour error by using the proposed methods for obtaining the mapping relation between the crankshaft pin journal contour error and the axis position control error. The mapping relation can be applied in predicting the key functional component performance of machine tools from the oscillating grinding workpiece contour error, which is of great significance for the automatic crankshaft production line, to improve efficiency and accuracy by selective inspection and monitoring.

## 2. Contour Error Decomposition Based on EEMD

The EMD, proposed by Huang et al. in 1998 [12], can decompose a complicated signal into a series of intrinsic mode functions (IMFs) based on the local characteristic time scale of the signal. An IMF satisfies the following two conditions: (1) The number of extrema and zero-crossings must be either equal or different at most by one; (2) At any point, the mean of the upper and lower envelopes from the signal is zero. If the *j*th IMF component is indicated as *C_j_*, then the original signal *X*(*t*) can be described as,

(1) $$X(t)=\sum_{j=1}^{n}C_{j}+r$$

where *r* is the residue of the signal and it is a monotonic function. In essence, EMD is an adaptive dyadic filter bank [16] which can decompose white noise into a series of IMF components with different center frequency [17]. In terms of practical application, as the measured data are not white noise, the mode mixing problem may appear. The result of mode mixing is that the IMF components lose physical significance of decomposition.

**Figure 2:**
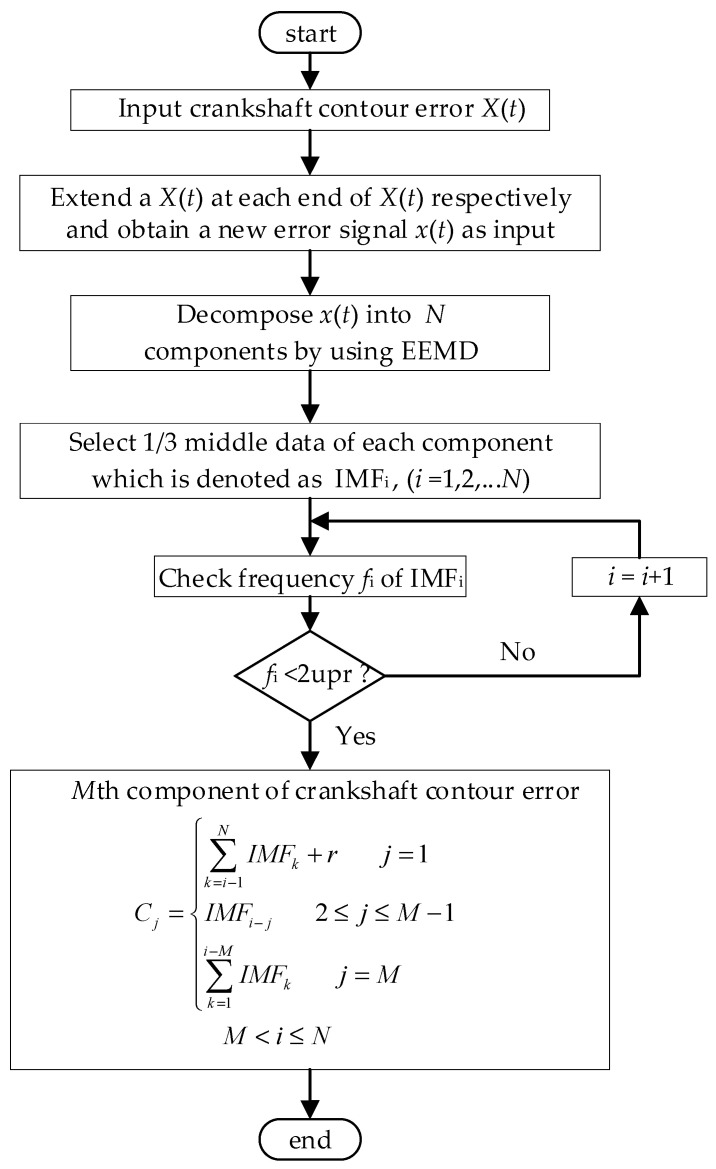
Decomposition process of the crankshaft pin journal contour error by EEMD.

To overcome the mode mixing problem in EMD, EEMD was proposed by Wu and Huang [13]. The decomposition of the crankshaft pin journal contour error based on EEMD is illustrated in Figure 2. The contour error represents closed data with the same head and tail. In order to avoid boundary effect, each end of contour error data is periodically extended with the same original contour error data. After EEMD decomposition, on third of the middle data from each component is selected as the decomposition result, then ranked from high frequency to low frequency. In theory, the contour error data does not include the 0 UPR (undulation per revolution) information which represents the dimension error information and 1 UPR information, which represents center deviation information of the crankshaft pin journal. Therefore, the lowest order component *C*_1_ of the pin journal contour error is composed of the previous order component, the components with frequency less than 2 UPR, and the residual component. If the contour error is decomposed into *M* components, $C_{j}$ corresponds to EEMD decomposition component in reverse order for the jth component $C_{j}$ (2≤j≤M−1). For the remaining high frequency components of EEMD decomposition, these components are combined into $C_{M}$ as the highest component of crankshaft contour error decomposition.

## 3. Effect of Axis Position Control Error on the Crankshaft Pin Journal Contour Error

**Figure 3:**
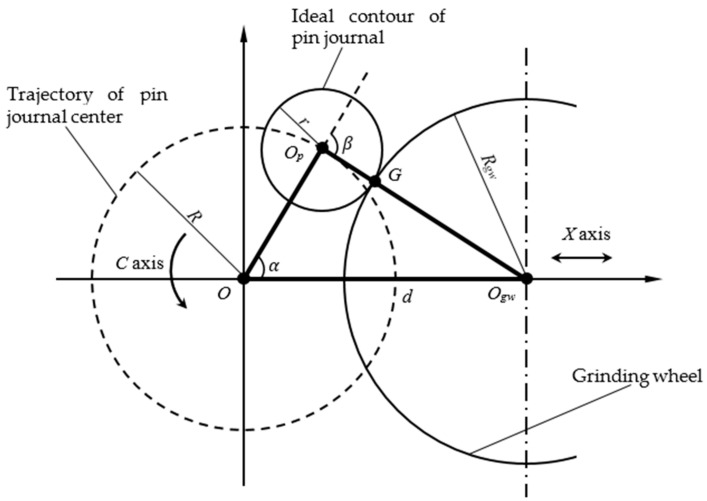
Principle diagram of crankshaft pin journal oscillating grinding under ideal conditions.

In the oscillating grinding process, the grinding wheel is always tangent with a crankshaft pin journal. The contour of crankshaft pin journal is produced by reciprocating motion of grinding wheel following rotational motion of crankshaft. Under ideal conditions, the contour of the crankshaft pin journal is a standard circle. The principle diagram of the crankshaft oscillating grinding motion is illustrated in Figure 3. The grinding point (tangent point) *G* is in the connecting line between the center of the pin journal $O_{p}$ and the center of grinding wheel $O_{gw}$. The crankshaft is driven under the workpiece driven by axis *C* and the pin journals rotate around the center *O* of the main journal. The grinding wheel implements reciprocating chasing motion along the X axis and realizes the grinding of crankshaft pin journals.

The contour control point (d,α) represents the motion control position information which determines the contour of the pin journal. This point maps the contour point (r,β) of the pin journal.

The contour control equation is,

(2) $$d=R\cos\alpha +\sqrt{{\left(r+R_{gw}\right)}^{2}-{\left(R\sin\alpha \right)}^{2}}$$

Equation (2) can be transformed as,

(3) $$r=\sqrt{{\left(d-R\cos\alpha \right)}^{2}+{\left(R\sin\alpha \right)}^{2}}-R_{gw}$$

According to the geometric relationship, Equation (4) can be achieved as,

(4) $$\beta =\alpha +\arcsin\frac{R\sin\alpha }{r+R_{gw}}$$

From Equations (3) and (4), Equation (5) can be obtained as follows,

(5) $$\beta =\alpha +\arcsin\frac{R\sin\alpha }{\sqrt{{\left(d-R\cos\alpha \right)}^{2}+{\left(R\sin\alpha \right)}^{2}}}$$

where d is the distance from the center of the grinding wheel $O_{gw}$ to the rotation center of the crankshaft O, with reciprocating motion of grinding wheel along X axis. R is the eccentric distance of the pin journal, which is the distance from center of the pin journal $O_{p}$ to the rotation center of the crankshaft O. r is the radius of the pin journal, which is the distance from the grinding point G to the center of the pin journal $O_{p}$. $R_{gw}$ is the radius of the grinding wheel. α is the angle between $OO_{p}$ and $OO_{gw}$, which is the rotation angle of the crankshaft controlled by C axis. β is the angle between the extended line of $OO_{p}$ and $O_{p}O_{gw}$, which is the angle corresponding to the grinding point passing through the arc.

When a workpiece is ground, the theoretical grinding motion control equations can be derived from Equation (2),

(6) α=α(t)

(7) $$d(t)=R\cos\alpha (t)+\sqrt{{\left(r+R_{gw}\right)}^{2}-{\left(R\sin\alpha (t)\right)}^{2}}$$

where R and r are the motion control parameters and they are constants. When a super hard grinding wheel is chosen, like the CBN grinding wheel, $R_{gw}$ may be considered as a constant because of the negligible wear.

According to Equations (6) and (7), the CNC system of the machine tool controls the motion of C axis and X axis to machine the pin journals. Only if the practical motion positions of C axis and X axis accurately meet the requirements of the equations, the grinding result is an ideal circle. However, in the practical machining, the motion control of the C axis and X axis both have errors. Therefore, the contour of the ground pin journal is not a standard circle.

**Figure 4:**
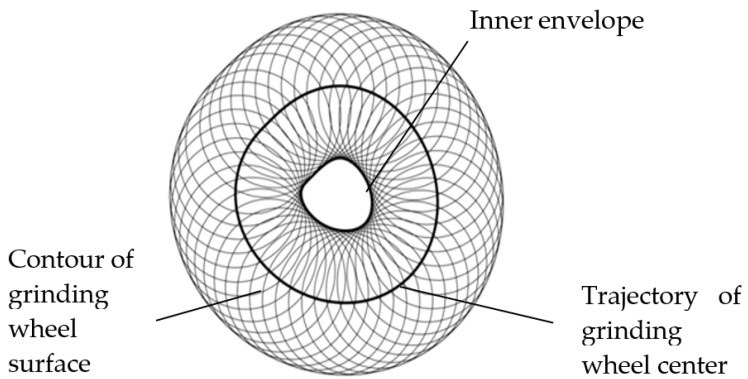
Schematic illustration of the grinding contour generation mechanism.

To accurately obtain the grinding contour generation mechanism of the crankshaft pin journal and the relation between the machine tool position control information and the crankshaft pin journal contour information, the coordinate system is created in the pin journal, where the grinding process can be considered as the grinding wheel rotation around the pin journal. If the elastic deformation of the grinding wheel and the workpiece is neglected, the inner envelope of the grinding wheel trajectory is the grinding contour of the pin journal, as illustrated in Figure 4.

**Figure 5:**
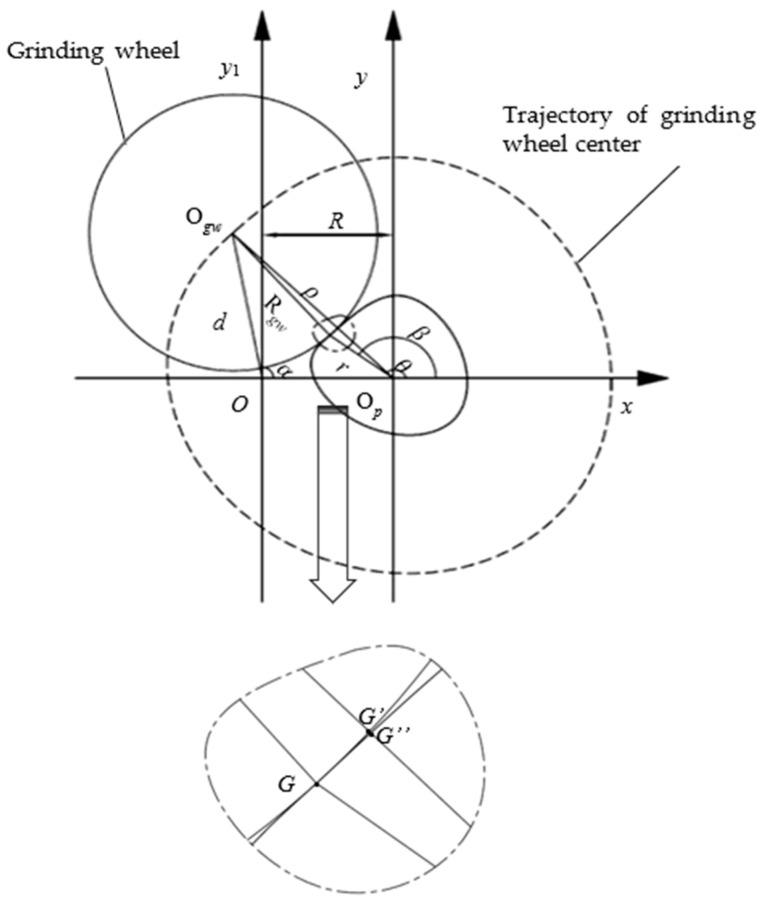
Principle diagram of the pin chasing grinding motion.

According to the above principle and neglecting elastic deformation of the mechanical system, the grinding wheel and workpiece utilize the practical contour control point (d,α), which is the practical coordinate position of grinder C axis and X axis, to interact for achieving crankshaft pin journal contour (r,β). The principle diagram of the pin chasing grinding motion is illustrated in Figure 5.

In the xOy_1_ coordinate system, the trajectory point of the grinding wheel center corresponds to the polar coordinate (d,α) and the rectangular coordinate (dcosα,dsinα). In the xO_p_y coordinate system, the grinding wheel center corresponds to the polar coordinate (ρ,θ) and the rectangular coordinate (dcosα−R,dsinα). Then, the following equations can be obtained,

(8) dcosα−R=ρcosθ

(9) dsinα=ρsinθ

According to Equations (8) and (9), Equations (10) and (11) can be obtained,

(10) $$\rho =\sqrt{{\left(d\cos\alpha -R\right)}^{2}+{\left(d\sin\alpha \right)}^{2}}$$

(11) $$\tan\theta =\frac{d\cos\alpha -R}{d\sin\alpha }$$

Let ρ(θ) denote the trajectory function of the grinding wheel center. According to the geometric relationship, the following equations can be obtained,

(12) $$r=\sqrt{{\left(\rho \cos\theta -R_{gw}\cos(\arctan\frac{\rho \sin\theta -\frac{d\rho (\theta )}{d\theta }\cos\theta }{\rho \cos\theta +\frac{d\rho (\theta )}{d\theta }\sin\theta })\right)}^{2}+{\left(\rho \sin\theta -R_{gw}\sin(\arctan\frac{\rho \sin\theta -\frac{d\rho (\theta )}{d\theta }\cos\theta }{\rho \cos\theta +\frac{d\rho (\theta )}{d\theta }\sin\theta })\right)}^{2}}$$

(13) $$\tan\beta =\frac{\rho \sin\theta -R_{gw}\sin(\arctan\frac{\rho \sin\theta -\frac{d\rho (\theta )}{d\theta }\cos\theta }{\rho \cos\theta +\frac{d\rho (\theta )}{d\theta }\sin\theta })}{\rho \cos\theta -R_{gw}\cos(\arctan\frac{\rho \sin\theta -\frac{d\rho (\theta )}{d\theta }\cos\theta }{\rho \cos\theta +\frac{d\rho (\theta )}{d\theta }\sin\theta })}$$

From Equations (12) and (13), the contour information (r,β) of the crankshaft pin journal with the crankshaft rotation center *O*, the practical pin journal center *O_p_* and the phase reference positive x axis direction can be inferred.

**Figure 6:**
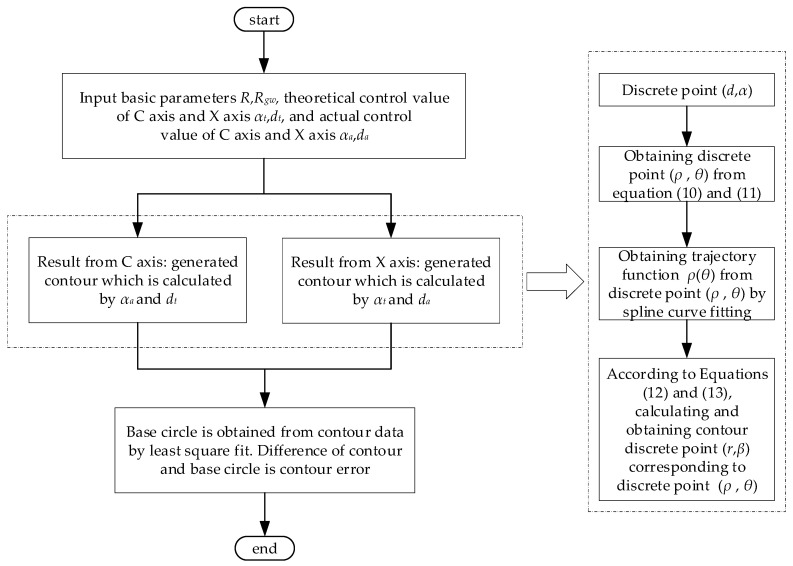
Calculation process of the crankshaft contour error caused by the axis position control error.

The base circle can be obtained from the crankshaft pin journal contour by least square fit. The difference of the practical contour and the base circle is the contour error [18]. In the position control, the deviation between the actual value and the theoretical value is inevitable. Therefore, the main factors that cause the contour error of the crankshaft pin journal root in the position control error of X axis and C axis. The basic geometric relationship is shown in Figure 5, and the principle of the pin chasing grinding motion relationship is illustrated by Equations (10)–(13). The calculation process of the crankshaft contour error caused by the axis position control error is described in Figure 6.

The axis position control error is the difference of the theoretical and the actual control value. If the basic machining parameters are known, the axis position control error is the equivalent information with the theoretical control value and the actual control value. To simplify the process, the theoretical control value and the actual control value of the axis can be directly acquired from the CNC system. The calculation of the effect of the C axis position control error on the crankshaft contour error is firstly conducted by the actual control value of the C axis and the theoretical control value of the X axis. Then, crankshaft contour information can be applied to calculate the contour error. Similarly, the effect of the X axis position control error on the crankshaft contour error can be obtained.

## 4. Grinding Experiments and Error Data Analysis

### 4.1. Grinding Experiments

**Figure 7:**
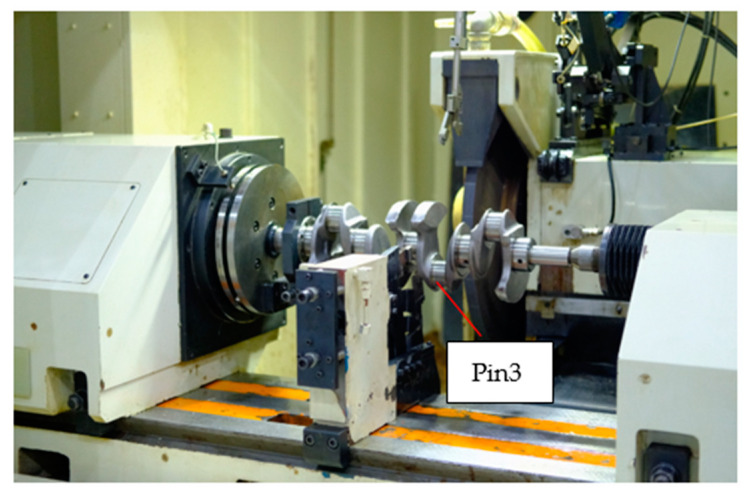
Setup of the grinding experiments.

**Figure 8:**
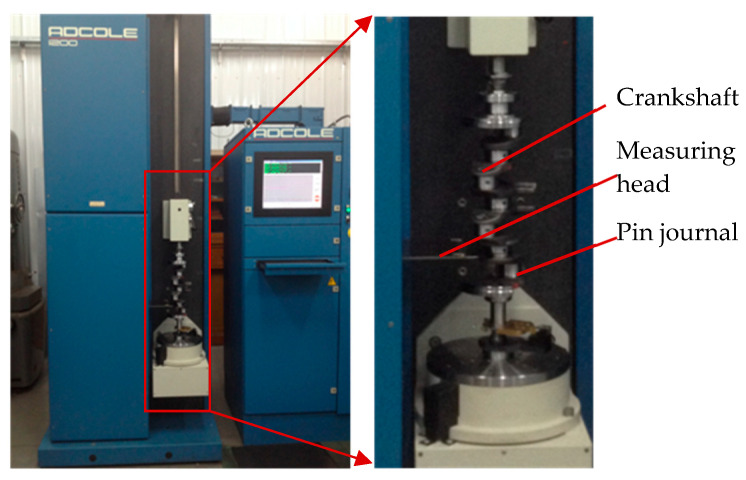
Measurement of the crankshaft pin journal contour error.

A CNC oscillating grinder is applied to machine the crankshaft pin journals, which is the H405-BF CNC crankshaft oscillating grinder developed by Shanghai Machine Tool Works Ltd. The setup of grinding experiments is shown in Figure 7 where Pin3 is ground. Many grinding experiments are done in this research. For validating the proposed methods above, a group of acquired and measured data is used. The CNC system is Siemens 840D. In the grinding process, the CNC system with trace function is utilized to acquire the synchronously position control errors of C axis and X axis. After finishing the grinding, the contour errors of the crankshaft pin journals are measured, as shown in Figure 8. The grinding wheel is a CBN grinding wheel with a radius of 299.970 mm. The crankshafts are from the L850 ECOTEC 2.4L engine production line of SAIC General Motors. The diameter of the pin journals is 48.040 mm and the eccentric distance is 49.000 mm.

**Figure 9:**
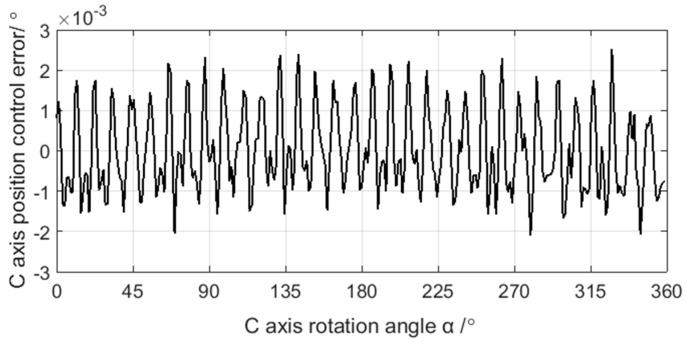
Acquisition data of the C axis position control error.

**Figure 10:**
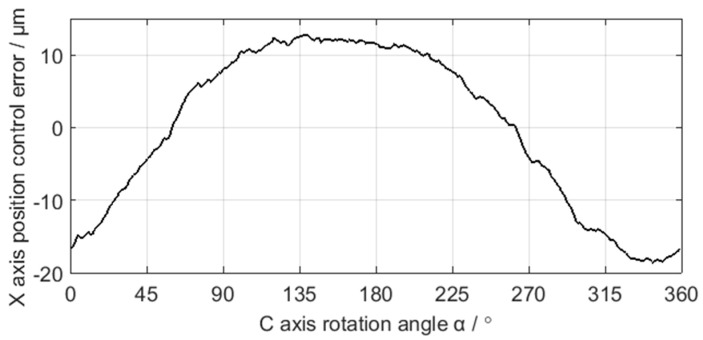
Acquisition data of the X axis position control error.

**Figure 11:**
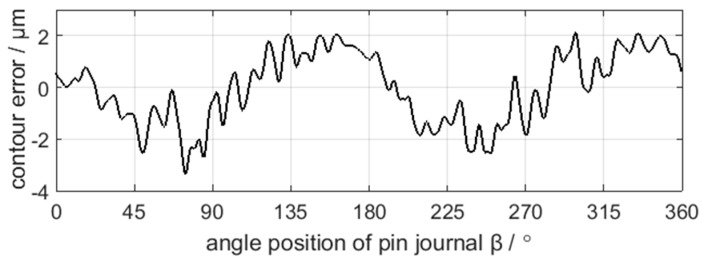
Measurement data of the crankshaft pin journal contour error.

The sampling period of C axis and X axis position control error is 4 ms, and the results are shown in Figure 9 and Figure 10. After grinding, the crankshaft is measured by the ADCOLE 1200SH crankshaft measuring machine to obtain the contour errors of the pin journals. The measurement data are filtered by a Gaussian filter with the cutoff frequency of 50 UPR. The contour of the pin journal consists of 1440 data points in 360° circumference, as shown in Figure 11.

### 4.2. Decompostion and Calculation Result of the Error Data

**Figure 12:**
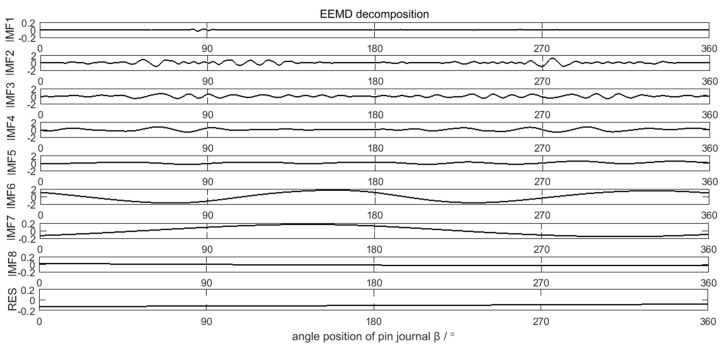
EEMD decomposition results of the crankshaft pin journal contour error.

**Figure 13:**
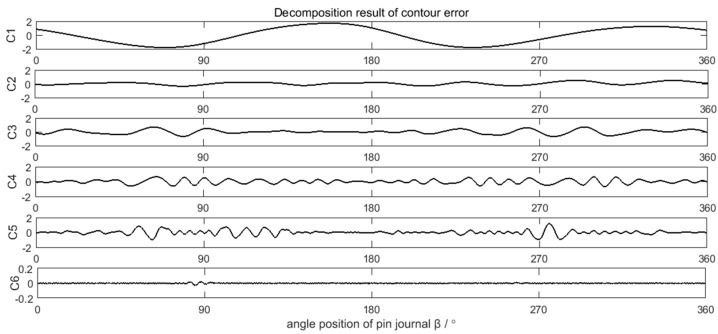
Decomposition result of the crankshaft pin journal contour error.

The measured crankshaft pin journal contour error in Figure 11 is decomposed by the EEMD method, as shown in Figure 12. The valid components in the EEMD decomposition results are IMF2–IMF8. IMF1 is the high frequency disturbance of the added white noise in EEMD decomposition process. According to the decomposition process in Figure 2, six decomposition components C1–C6 of the crankshaft pin journal contour error are obtained, as shown in Figure 13.

**Figure 14:**
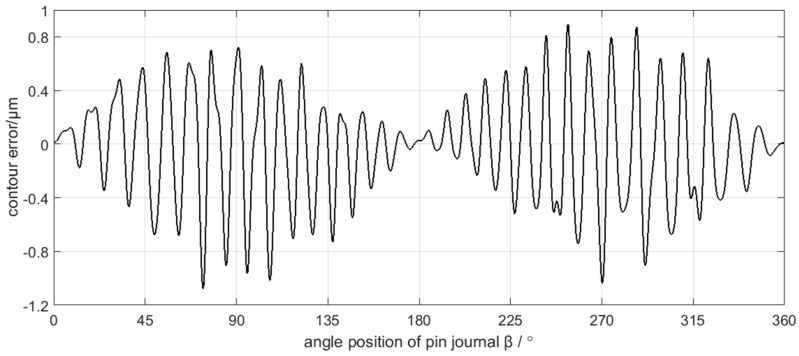
The calculated contour error of the pin journal caused by C axis position control error.

**Figure 15:**
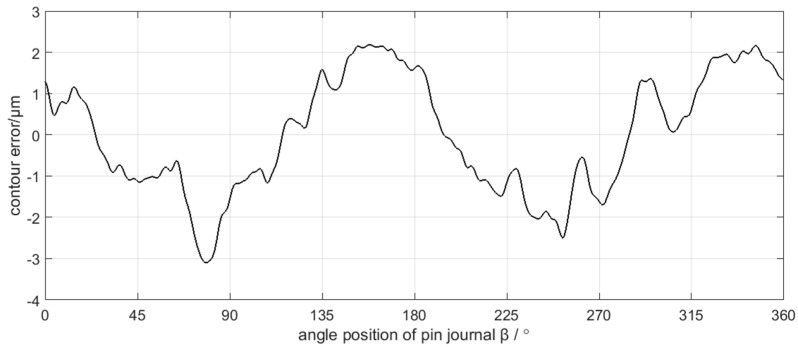
The calculated contour error of the pin journal caused by X axis position control error.

Neglecting the deformation of the grinding carriage, the grinding wheel, and the grinding wheel spindle, the position control information of C axis and X axis is equivalent to the trajectory information of the grinding wheel center. According to the grinding contour generation mechanism of the crankshaft pin journal, the workpiece contour can be obtained from the trajectory of the grinding wheel center. As the measured contour error of the crankshaft pin journal uses Gaussian filtering with a 50 UPR cutoff frequency, the same filtering processing is applied in the contour error calculation from the axis position control error for better comparability. The calculated contour errors of the crankshaft pin journal caused by the C axis position control error and X axis position control error are shown in Figure 14 and Figure 15, respectively.

### 4.3. Discusstion

**Figure 16:**
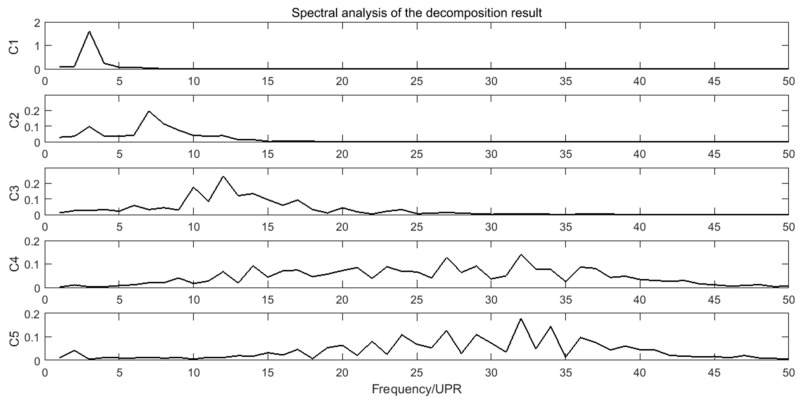
Spectra of C1 to C5.

**Figure 17:**
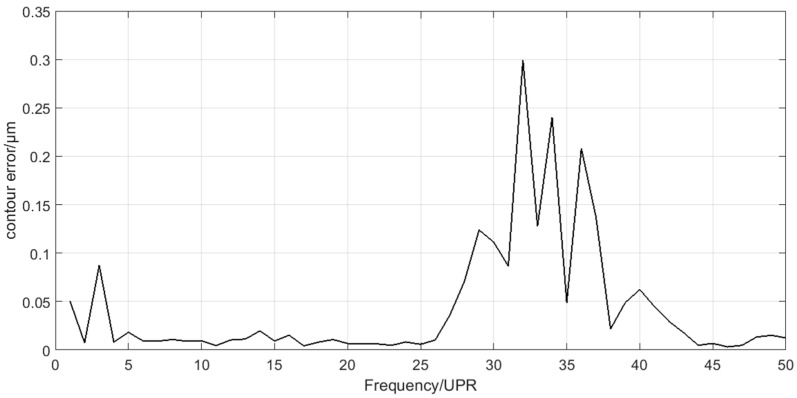
Spectrum of the contour error caused by C axis position control error.

**Figure 18:**
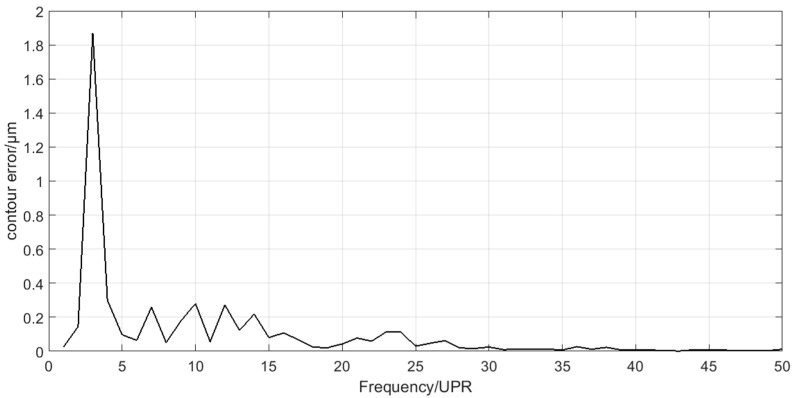
Spectrum of the contour error caused by X axis position control error.

Spectra analysis can be applied in the decomposition results of the crankshaft pin journal contour error in Figure 13. The amplitude of C6 component is very small, which can be neglected. The spectra of C1–C5 are shown in Figure 16. Figure 16 shows that the main frequency components of C1 to C3 are less than 15 UPR and the main frequency components of C4 to C5 are greater than 25 UPR. The calculation results of the crankshaft pin journal contour error caused by the axis position control error in Figure 14 and Figure 15 can also be analyzed in frequency domain. The spectra analysis results are shown in Figure 17 and Figure 18. Figure 15 and Figure 18 show that the main frequency components of the crankshaft pin journal contour error caused by X axis position control error are less than 15 UPR. Figure 14 and Figure 17 show that the main frequency components of the crankshaft pin journal contour error caused by C axis position control error are greater than 25 UPR.

**Figure 19:**
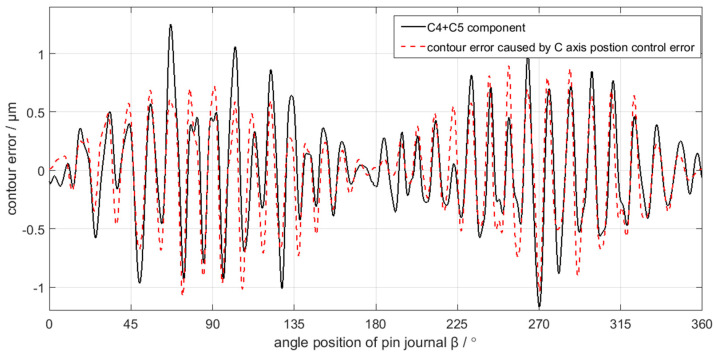
C4 + C5 component mapping into contour error caused by C axis position control error.

**Figure 20:**
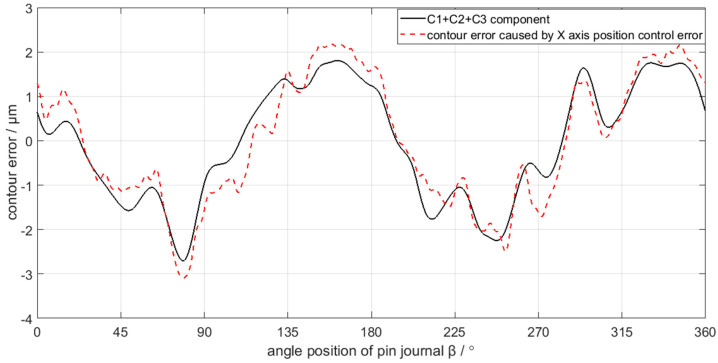
C1 + C2 + C3 component mapping into contour error caused by X axis position control error.

In comparison, C4 and C5 maps into contour error caused by C axis position control error, while C1, C2, and C3 correspond to the contour error caused by X axis position control error. The mapping relation can be validated in Figure 19 and Figure 20, where C4 and C5 is nearly the same with the contour error caused by C axis position control error, while C1, C2, and C3 are also nearly the same as the contour error caused by the X axis position control error. Therefore, the decomposition and calculation method proposed in this paper can effectively find out the mapping relation between the contour error components of the crankshaft pin journal and the axis position control error of the crankshaft oscillating grinder.

On the one hand, the mapping relation shows that we can predict the performance of the C axis and X axis through the contour error analysis of the crankshaft pin journal. On the other hand, it shows that the position control error of C axis and X axis must result in the contour error of the machined workpiece. Moreover, the contour error spectra caused by the C axis and X axis position control error in Figure 17 and Figure 18 show that the frequency components are separated. If they are mixed together, the EEMD method may not be applicable.

Although Figure 19 validates the mapping relation between the C4 and C5 components of the crankshaft pin journal contour error and the C axis position control error, there are still some minor differences in magnitudes of the contour error, which are more obvious in some peaks and valleys of the contour error waveform. The difference is less than 0.1 μm on average and less than 0.5 μm on the maximum. Similarly, in Figure 20, there are still some minor differences in the mapping relation between the C1 to C3 components and the X axis position control error. The difference is less than 0.5 μm on average and less than 1 μm on the maximum. The reason of causing the minor difference is the contour error of the crankshaft pin journal does not completely root in the position control error of the C axis and X axis. There are other error sources that affect the contour error of the crankshaft pin journal, but the position control error of the C axis and X axis constitute the main influence factors. Therefore, the mapping relation between the contour error of the crankshaft pin journal and the axis position control error has engineering application value.

## 5. Conclusions

(1) The mapping relation between the contour error components of the crankshaft pin journal and the axis position control error of the oscillating grinder is obtained. Because there are other error sources that affect the contour error of the crankshaft pin journal, there are some minor differences in the magnitude of the contour error. However, the position control error of the C axis and X axis represent the main influencing factors.
(2) A contour error decomposition method based on EEMD is proposed. Boundary periodic extension is applied to avoid the boundary effect and the low frequency component discrimination method is set up to effectively extract the low frequency components.
(3) The mapping relation between the contour error components of the crankshaft pin journal and axis position control error of the oscillating grinder can be applied in predicting the key functional component performance of machine tools from oscillating grinding workpiece contour error.

## Data Availability

The Excel-file data of the crankshaft pin journal contour errors and the axis position control errors used to support the findings of this research is available from the corresponding author upon request.
